# Effects of selfing and outcrossing on transgenerational responses to predation risk

**DOI:** 10.1007/s00442-025-05729-w

**Published:** 2025-05-30

**Authors:** Haley R. Altadonna, Lynne E. Beaty

**Affiliations:** School of Science, Penn State Erie, The Behrend College, Erie, PA 16563 USA

**Keywords:** Anti-predator behavior, Intergenerational effects, Morphometrics, Plasticity, Reproductive strategy, Self-fertilization

## Abstract

Phenotypic plasticity, an organism’s ability to change traits in response to its environment, can improve an individual's fitness by promoting a phenotype better suited for current environmental conditions. Phenotypic expression can be altered by direct experiences (i.e., within-generation plasticity) or experiences of previous generations (i.e., transgenerational plasticity). Transgenerational plasticity can help offspring overcome environmental stressors, such as predation, using epigenetic information from their parent(s). Offspring typically receive information from two parents though simultaneous hermaphrodites can produce offspring with varied contributions of epigenetic information by reproducing via outcrossing or self-fertilization (i.e., selfing). While the impact of predation on transgenerational effects has been described, the relative influence of epigenetic information from one or two parental sources is unknown. This study aimed to determine how transgenerational epigenetic effects of predation risk are influenced by selfing and outcrossing. Physid snails were collected and reared over two generations to evaluate anti-predator behavior and shell morphology. The F_1_ generation was exposed to treatments consisting of all combinations of predation risk and a mate's absence or presence, resulting in six F_2_ generation treatment lineages. Shell morphometrics and a behavioral assay were completed to quantify the anti-predator response of the F_2_ generation. We found that the offspring of outcrossers were larger, and their size was influenced by paternal predator treatment. F_2_ snails were also less likely to exhibit anti-predator behavior after exposure to predation risk. These findings provide insights into our understanding of transgenerational effects in species with various reproductive strategies.

**Supplementary Information:**

The online version contains supplementary material available at 10.1007/s00442-025-05729-w.

## Introduction

Phenotypic plasticity, an organism’s ability to change traits in response to its environment, can improve an individual's fitness by promoting a phenotype better suited for current environmental conditions. It can occur by direct exposure to environmental conditions (i.e., within-generation plasticity; West-Eberhard 2003), or by environmental conditions experienced by previous generations (i.e., transgenerational plasticity; Agrawal et al.^1999^, Galloway and Etterson 2007). Widespread across plant and animal taxa (Jablonka and Raz 2009), transgenerational plasticity (TGP) of various behavioral and morphological traits has been observed in response to abiotic (e.g., temperature, Salinas and Munch 2011) and biotic (e.g., predation, Coslovsky and Richner 2011) stressors. In many cases, TGP may be advantageous by allowing offspring to match their phenotype to past environmental conditions (Uller 2008). Thus, transgenerational plasticity can serve as an adaptive mechanism for offspring to cope with environmental variation by utilizing information cues passed on by their parent(s) (Agrawal et al.^1999^; Galloway and Etterson 2007).

Environmental variation in predation risk is known to illicit plastic responses in prey. Within a generation, prey may alter their behavior or morphology in response to predation risk (Lima and Dill 1990; Bourdeau and Johansson 2012) though there is evidence that these plastic responses can be produced across generations as well (Agrawal et al. 1999; Coslovsky and Richner 2011; Beaty et al. 2016; Tariel et al. 2020a, b). Much of the research on predator-induced TGP has focused on the effects of the parental environments and discerning the interactions between parental and personal environments (e.g., Tariel et al. 2020a, b). Ultimately, the integration of this information can be influenced by many factors. For example, sexually produced offspring typically receive both maternal and paternal information cues, which may illicit different maternal and paternal effects on the offspring’s phenotype (i.e., sex-specific transgenerational plasticity; Embroski and Mikheyev 2019, Gilad and Scharf 2019; Burke et al. 2020). Additionally, maternal and paternal information cues may be perceived by offspring differently due to ecological differences between sexes, which can lead to differences in information reliability (Kamel et al. 2010; Burke et al. 2020). Consolidating maternal and paternal cues can become complicated if there is a large amount of conflicting information, especially when also accounting for current environmental cues. This process may be further complicated by a species mating system or reproductive strategy.

A species’ reproductive strategy (e.g., sexual or asexual reproduction) determines how genetic information is passed to offspring and often promotes the goal of maximized fitness for the species (Goodwillie et al. 2005; Jarne and Auld 2006; Escobar et al. 2009). This can also influence information certainty as it applies to the adaptive benefits of phenotypic plasticity as the mating strategy of the parent(s) may influence the reliability of information cues. For example, offspring produced via self-fertilization may experience greater information certainty, as they receive information cues from only one parent. Many simultaneous hermaphrodites can produce female and male gametes at any given time, providing the option to reproduce via outcrossing or self-fertilization (i.e., selfing; Jarne and Auld 2006).

The ability to reproduce via outcrossing or selfing presents a unique genetic trade-off for simultaneous hermaphrodites. It has been hypothesized that organisms may choose to self-fertilize due to environmental stressors (e.g., mate scarcity or predation risk; Auld et al. 2016). Despite the potential short-term advantage this may offer, there are several long-term issues associated with selfing, mainly inbreeding depression, a decrease in fitness of self-fertilized offspring in comparison to cross-fertilized offspring (Charlesworth and Charlesworth 1987). Inbreeding depression is attributed to increased homozygosity and generally associated with lower fertility, survival, and growth rates (Charlesworth and Hughes 1996; Hoare and Hughes 2001). In contrast, outcrossed individuals may experience heterosis, an increase in fitness of outcrossed offspring compared to self-fertilized offspring, which is often attributed to increased heterozygosity reducing the effects of deleterious alleles (Escobar et al. 2008). Outcrossing offers a long-term adaptive advantage by increasing the efficiency of genetic recombination (Lande and Porcher 2015), resulting in increased genetic variation (Glemin and Ronfort 2013). Increased genetic variation may influence a population’s ability to adapt to environmental conditions, with outcrossing populations more likely to overcome such challenges (Noël et al. 2017).

Previous studies have examined the within-generation and transgenerational plasticity of anti-predator behavior and morphology in the simultaneous hermaphrodite *Physa acuta* (e.g., Beaty et al. 2016; Goeppner et al. 2020; Tariel et al. 2020a, b). *Physa* are known to induce within-generational plasticity in response to predation risk by altering shell shape (e.g., elongated, DeWitt et al. 2000; narrow aperture, Alexander and Covich 1991), and escape behavior (e.g., crawling-out of the water, Alexander & Covich 1991). Similarly, transgenerational plasticity of shell morphology and escape behavior have been observed in *Physa* (Beaty et al. 2016; Goeppner et al. 2020; Tariel et al. 2020a, b). However, it is unknown how reproductive strategy (i.e., selfing or outcrossing) of parents influences the strength of transgenerational effects in offspring. One could predict that the strength of transgenerational effects may vary between outcrossed offspring versus selfed offspring, with potentially stronger effects in selfed offspring due to greater information certainty. Here, we aimed to determine how selfing and outcrossing influence transgenerational epigenetic effects of predation risk in *Physa*. Specifically, we aimed to address the following questions: 1. How does predation risk and reproductive strategy of the F_1_ generation of snails impact their shell morphology? 2. How does predation risk and reproductive strategy of the F_1_ generation impact their reproductive output? 3. How does reproductive strategy and exposure to predation risk of the F_1_ generation influence the shell morphology of the F_2_ generation? and 4. How does reproductive strategy and exposure to predation risk of the F_1_ generation influence the anti-predator behavior of the F_2_ generation?

## Methods

### Animal collection

We collected 120 wild F_0_ generation Physid snails by hand from Fourmile Creek in Erie County, Pennsylvania (42°06ʹ52.4″N 79°59ʹ22.3″W) on 29 June 2022. Snails were transferred to laboratory facilities within an hour of capture and housed in a 3 L plastic enclosure filled with 2 L dechlorinated water. After five days, the adults were removed from the enclosure, and the deposited egg masses were allowed to hatch (see Fig. S1 for a complete graphical timeline and experimental design). After developing for seven days, 120 of the resulting F_1_ snails were removed and housed individually in 59 mL plastic containers filled with 50 mL of dechlorinated water.

### F_**1**_ predator treatments

The F_1_ snails were exposed to either predator cues (P, *n* = 60) or dechlorinated water-only control cues (C, *n* = 60) three times weekly to determine the impact of predation risk on the F_1_ and F_2_ generations. Predation risk can influence shell morphology of the exposed generation as *Physa* exposed to predator cues have developed elongated shells (Dewitt et al. 2000) or more narrow apertures (Alexander and Covich 1991). Predator-exposed snails may also produce larger offspring (Beaty et al. 2016), and these offspring may show plasticity in their anti-predator escape behaviors (Beaty et al 2016; Luquet and Tariel 2016).

We created the predator cues by placing one wild-caught Rusty crayfish (*Orconectes rusticus*) into a 1.7 L glass container filled with 600 mL dechlorinated water and feeding it 1.5 g of live *Physa* snails collected from a pond on Penn State Behrend’s campus in Erie County, Pennsylvania (42°07ʹ33.8″N 79°58ʹ25.7″W). After one hour, we removed the water from the crayfish container and combined it with a solution of 1.5 g of live *Physa* snails crushed in 200 mL dechlorinated water. Previous studies have shown that *Physa* exhibit anti-predator responses to the combination of cues of *Procambarus simulans* fed snails and crushed conspecifics (Alexander and Covich 1991). We strained the predator cue solution and froze it in 2 mL aliquots. Dechlorinated water was also frozen in 2 mL aliquots to be used as control cues. Both cues were thawed and homogenized in batches three times weekly during water changes. Before adding predator or control cues, we changed 50% of the water in each snail housing container and fed them crushed Omega One veggie rounds ad libitum. We then added 1 mL of predator cue or control cue to each container.

### F_**1**_ reproduction treatments

After four weeks of predator treatments (i.e., approximately five weeks post-hatch), we placed all F_1_ snails in containers with 50 mL predator-free dechlorinated water to prevent egg masses from being exposed to predator cues. The F_1_ predator treatments were combined with reproduction treatments of either selfing (S, *n* = 40) or outcrossing (O, *n* = 80), resulting in a full-factorial design (F_1_ Reproduction treatment-F_1_ predator treatment: S-C, S-P, O-C, O-P). Outcrossing snails may experience differences in shell morphology due to the act of intromission. Additionally, selfers may be larger compared to outcrossers if reproduction is delayed and resources are instead allocated toward growth (Tsitrone et al. 2003). Preferentially outcrossing snails also experience reduced fecundity when isolated from a mate (Jarne et al. 1991, 2000), and selfing snails may produce fewer fertile eggs and less viable offspring than outcrossing counterparts (Wethington and Dillon 1997). It was expected that the reproduction treatments would impact the shell morphology and the anti-predator behavior of the F_2_ generation as the amount and the certainty of information will vary between offspring. Reproduction treatments occurred twice weekly for three hours each time. Prior to reproduction treatments, each snail was marked with a nail polish color unique to their treatment. During reproduction treatments, all F_1_ snails were temporarily transferred to new 88 mL containers filled with 75 mL of predator-free dechlorinated water (i.e., mating containers). In these mating containers, selfing snails were isolated from a mate, and outcrossing snails were randomly paired with a mate. We paired outcrossing snails into four groups based on predator treatment (F_1_ maternal predator treatment-F_1_ paternal predator treatment: C–C, C-P, P–C, P-P). *Physa* preferentially outcross (Jarne et al. 2000), so all egg masses obtained from these pairings were treated as outcrossed offspring. Maternity was indicated by the predator treatment of the snail that laid the eggs, and paternity was indicated by the predator treatment of the snail that was randomly paired with it.

After three hours, snails were returned to their original cups, which were checked daily for deposited egg masses for six weeks. Each day, we removed the egg masses from the parental cup and recorded the number of egg masses deposited by each snail and the number of eggs in each mass. Snails may employ different reproductive strategies in response to predation risk by altering the number of egg masses they produce, or the number of eggs per mass. That is, snails may opt to produce more egg masses with fewer eggs per mass or reduce overall reproductive investment to increase growth. We also recorded the number of blank eggs (i.e., eggs without a developing embryo) and the number of deformed eggs (i.e., unviable) in each egg mass. Selfers typically produce fewer eggs compared to outcrossers and have a larger proportion of blank or otherwise unviable eggs (Wethington and Dillon 1997). This may further be impacted by the F_1_ predator treatment as reduced fecundity is a common response to predation risk (Magnhagen 1991). The F_2_ egg masses were then placed into one of six lineage-specific housing containers based on F_1_ treatments (F_1_ reproduction treatment-F_1_ maternal predator treatment + F_1_ paternal predator treatment: S-C, S-P, O-C + C, O-C + P, O-P + P, O-P + C). Each lineage's eggs were placed into separate 3 L plastic enclosures filled with 2 L predator-free dechlorinated water. After the six-week reproduction period, 20 F_2_ snails from each lineage were removed and housed individually in 59 mL plastic containers filled with 50 mL of dechlorinated water.

### F_**1**_ and F_**2**_ shell morphology

We quantified the shell shape and the size of all adult F_1_ and F_2_ snails using geometric morphometrics (Zelditch et al. 2004). The F_1_ snails were measured immediately following the end of the reproduction treatment period to determine the influence of the predator and reproduction treatments on parental shell size and shape. The F_2_ snails were measured approximately six weeks post-hatch and prior to the following behavioral assay. We photographed each snail next to a ruler with their aperture up using a Leica EZ4 HD dissecting microscope connected to Leica LAS software (version 4.1). Following Beaty et al. (2016) and Goeppner et al. (2020), we digitized 11 homologous landmarks and 17 semi-landmarks on images of each snail with the program tpsDIG (Rohlf 2013). For each generation, we used the program tpsRelW (Rohlf 2010) to perform a Procrustes analysis that calculated relative warps (RWs); i.e., axes that summarize variation in shell shape). We then calculated centroid size (i.e., the square root of the sum of distances squared from each landmark to the centroid of the landmark) as a metric for shell size.

### F_**2**_ anti-predator behavior

We placed each F_2_ snail into a 450 mL plastic behavioral arena filled with 350 mL dechlorinated water. We allowed the snails to acclimate for one hour, then placed sinking algae disks into each arena to incentivize them to move to the bottom of the cup. *Physa* are known to respond to crayfish predators by crawling out of the water as moving to the bottom of the cup would be dangerous in their presence (Alexander and Covich 1991, McCarthy and Fisher 2000). Alternatively, *Physa* may increase refuge use (Turner 1996) or bury into the substrate (Snyder 1967) when exposed to water with crushed conspecifics. We scored snails at or above the waterline as exhibiting anti-predator behavior (i.e., crawling out). We first added 1 mL of water-only control cue to each arena and recorded the position of each snail relative to the water line every 15 min for 2.5 h. We then added 1 mL of predator cue to each arena and repeated the observation procedure.

### Analysis

To evaluate the effect of treatments and time on F_1_ reproductive strategy, we conducted model selection analysis using Akaike’s Information Criterion corrected for small sample sizes (AICc) and evaluated relative support for candidate linear mixed models (LMMs) or generalized linear mixed models (GLMMs). With either the number of egg masses, number of eggs per mass, proportion of blank eggs per mass, or proportion of deformed eggs per mass produced as response variables, we constructed models that contained all combinations of F_1_ predator treatment, F_1_ reproduction treatment, and days since egg production as fixed factors. Individual snail was treated as a random factor for all analyses. We also constructed and compared linear models examining the impacts of F_1_ predator treatment and F_1_ reproduction treatment on the total number of eggs produced over the course of the experiment to investigate treatment effects on reproductive output.

We again used model selection analyses to determine the effect of F_1_ treatments on F_1_ and F_2_ snail size (i.e., centroid). For each generation, we constructed alternative linear models (LMs) with centroid as the response variable and F_1_ predator and reproduction treatments as explanatory factors. For the models of F_2_ snail size, we included maternal and paternal predator treatments as separate factors in the analysis.

The effect of treatments on shell shape for the F_1_ and F_2_ generation was evaluated using mixed MANCOVAs in the MIXED package of SAS following Riesch et al. (2013) and Goeppner et al. (2020). We used the first 20 RWs (describing at least 95% of the shape variation in the snails) as dependent variables, which were treated as repeated measures for each snail, and centroid, F_1_ predator treatment, and F_1_ reproduction treatment as independent variables. The *p* values for the interaction between the RWs and the independent variables were calculated using the Kenward–Rogers approximation of degrees of freedom and used to determine whether snail size, F_1_ reproduction treatment, and F_1_ predator treatment significantly affected the RWs.

To visualize differences in shape between F_1_ and F_2_ snails from different F_1_ predator and reproduction treatments, we calculated divergence vectors (DV; Langerhans 2009) that showcased the maximum differences in shape between treatments. The DV values were not used in our analyses to determine how treatments influenced shape; they were used for plotting purposes only.

To evaluate F_2_ anti-predator behavior, we conducted model selection analysis between candidate generalized linear mixed models (GLMMs) with a binomial link function that contained all combinations of F_1_ reproduction treatment, F_1_ predator treatment, and the presence or absence of predator cues in the behavioral arena as fixed factors. Whether or not the snail was exhibiting anti-predator behavior (i.e., had crawled out of the water = 1, or remained in the water = 0) was the response variable. Individual snail was treated as a random factor.

We constructed all LMs, LMMs, and GLMMs using either the “lme4” package (Bates et al. 2015) or “lmerTest” package (Kuznetsova et al. 2017) in R version 4.3.0 (R Core Team 2022) and compared the models using Akaike’s Information Criterion corrected for small sample sizes (AICc). Models were selected based on the lowest relative AICc score such that the model with the lowest AICc was viewed as being the most supported (Anderson 2007). Models that were < 2 AICc from the lowest model were considered equally supported.

## Results

Given the length of our experiment and the small size at which snails were initially introduced to treatments, some mortality occurred across all treatments. As a result, our final sample sizes for the F_1_ generation were (out of the original 20 for each S and 40 for each O): S-C = 18, S-P = 19, O-C = 37, O-P = 39. The final sample sizes for our F_2_ generation were (out of the original 20 for each): S-C = 18. S-P = 6, O-C + C = 17, O-C + P = 9, O-P + C = 18, O-P + P = 18.

### F_**1**_ reproduction

**Fig. 1:**
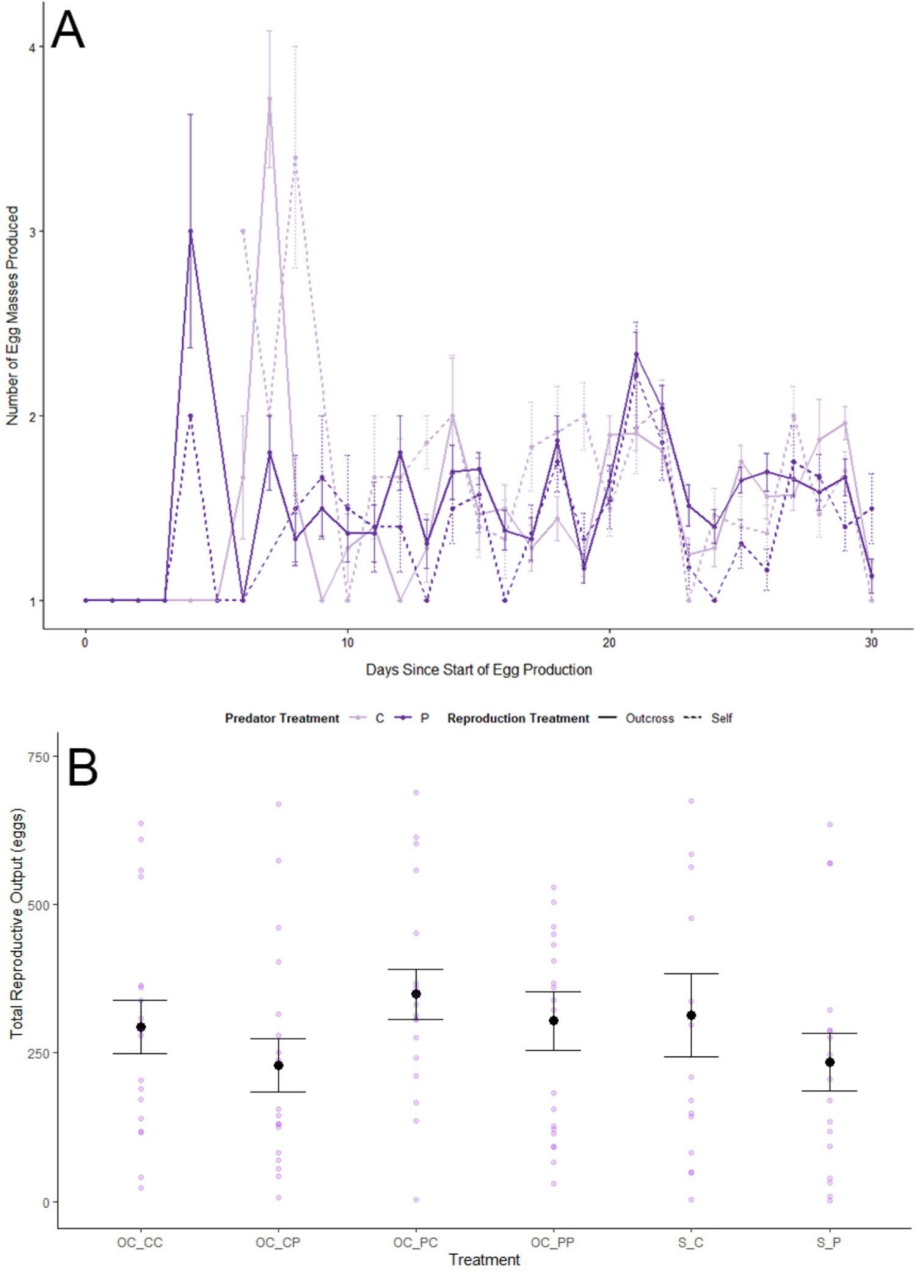
**A** Mean number of egg masses produced daily ± SE by F_1_ snails (*Physa sp.*) since the beginning of egg production. Reproduction treatments: outcrossing (solid), selfing (dotted). Predator treatments: water-only control cues (light purple), predator cues (dark purple). **B** Mean total reproductive output by snails ± SE over the course of the experiment in different F_1_ reproduction (OC = outcrossing, S = selfing) and F_1_ predator treatments (P = predator cues, C = water-only control cues). In the outcrossing treatments, the first F_1_ predator cue letter corresponds to the treatment that the individual snail received, and the second letter is the predator treatment that their partner received. The transparent purple points indicate individual snail data. Snail sample size by treatment combination: outcross-control (n = 36), outcross-predator (*n* = 38), selfing-control (*n* = 15), selfing-predator (*n* = 17)

F_1_ predator and reproduction treatments influenced some aspects of reproductive success. The total number of egg masses produced by each group was: O-C + C = 523, O-C + P = 364, O-P + C = 582, O-P + P = 557, S + C = 405, and S + P = 335. There were two supported models to explain the impact of F_1_ treatments and time on the number of egg masses produced—the model containing F_1_ predator treatment interacting with time and the model containing F_1_ reproduction treatment interacting with time (Table 1, Fig. 1). Predator-treated snails produced fewer egg masses earlier than control-treated snails (mean ± SE; Predator: 1.63 ± 0.02 masses/snail/day, Control: 1.70 ± 0.03 masses/snail/day), and outcrossing snails produced more egg masses earlier than selfing snails (Outcross: 1.68 ± 0.02 masses/snail/day, Self: 1.62 ± 0.03 masses/snail/day). The total number of eggs produced by each group was: O-C + C = 5286, O-C + P = 4131, O-P + C = 6195, O-P + P = 6166, S + C = 4699, and S + P = 3991. The number of eggs produced per egg mass increased over time but was not influenced by the F_1_ predator or reproduction treatments (Table 1). The total number of deformed eggs produced by each group was: O-C + C = 29, O-C + P = 38, O-P + C = 38, O-P + P = 43, S + C = 37, and S + P = 23. The total number of blank eggs produced by each group was: O-C + C = 3, O-C + P = 0, O-P + C = 2, O-P + P = 6, S + C = 11, and S + P = 8. While there was some evidence that the proportions of blank and deformed eggs were impacted by day and F_1_ reproduction treatment, the null model was equally supported (Table 1).

**Table 1:** Model selection results for F_1_ reproduction based on combinations of F_1_ predator treatment (Pred; predator or control), and F_1_ reproduction treatment (Repro; selfing or outcrossing) over time (Day), if applicable

	ΔAICc	Df	Weights
Number of egg masses models			
Pred*Repro*Day	0	14	0.38
Repro*Day	0.4	6	0.31
Pred*Day	1.1	6	0.22
Day	3.1	4	0.08
Null	8.5	3	< 0.01
Repro	9.4	4	< 0.01
Pred	10.0	4	< 0.01
Number of eggs per egg mass models			
Day	0.0	4	0.57
Repro*Day	2.8	6	0.14
Pred*Day	3.0	6	0.13
Null	3.7	3	0.09
Repro	5.7	4	0.03
Pred	5.7	4	0.03
Pred*Repro*Day	9.8	14	< 0.01
Proportion of blank eggs models			
Repro	0.0	3	0.34
Repro*Day	0.9	5	0.22
Null	1.0	2	0.21
Day	2.5	3	0.10
Pred	2.8	3	0.09
Pred*Day	4.7	5	0.03
Pred*Repro*Day	13.6	14	< 0.001
Proportion of unviable eggs models			
Repro*Day	0.0	5	0.28
Null	0.4	2	0.24
Repro	0.8	3	0.19
Day	1.3	3	0.15
Pred	1.9	3	0.11
Pred*Day	4.9	5	0.02
Pred*Repro*Day	7.5	13	< 0.01
Total reproductive output			
Null	0.0	2	0.50
Repro	1.9	3	0.20
Pred	1.9	3	0.20
Repro*Pred	3.1	3	0.10

* Interaction terms are indicated with asterisks

For each model, except for the models examining total reproductive output, individual snail was treated as a random effect

### Shell size and shape

**Fig. 2:**
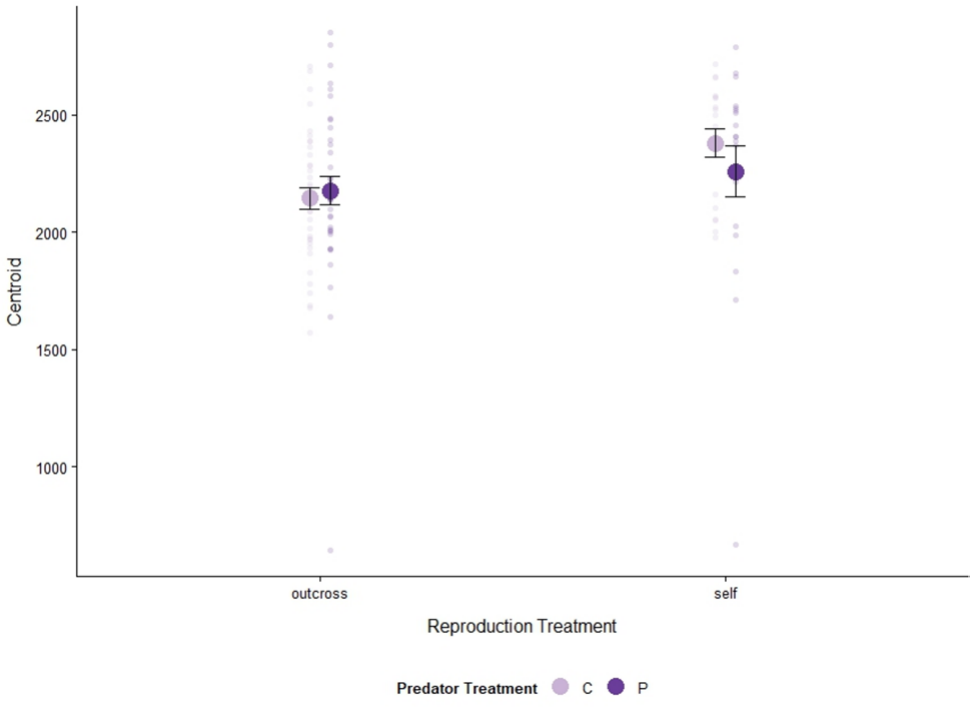
Mean shell size (centroid size ± SE) of snails from F_1_ treatments. Predator treatments: water-only control cues (light purple), predator cues (dark purple). Sample size by treatment combination: outcross-control (*n* = 37), outcross-predator (*n* = 39), selfing-control (*n* = 18), and selfing-predator (*n* = 19)

**Fig. 3:**
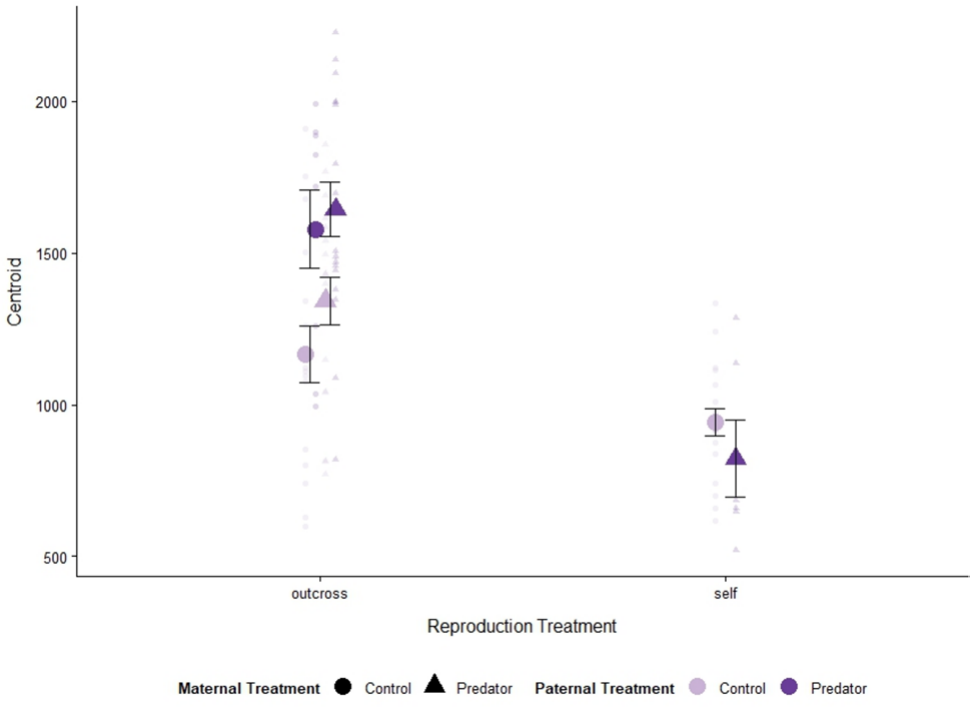
Mean shell size (centroid size ± SE) of snails from each F_2_ reproduction treatment. Maternal predator treatments: water-only control cues (circle), predator cues (triangle). Paternal predator treatments: water-only control cues (light purple), predator cues (dark purple). The transparent purple points indicate individual snail data. Sample size by treatment combination, where treatment combination is indicated as F_1_ reproduction treatment (S = selfing, OC = outcrossing)-F_1_ maternal predator treatment + F_1_ paternal predator treatment (C = control cues, P = predator cues): OC-C + C (*n* = 17), OC-C + P (*n* = 9), OC-P + C (*n* = 17), OC-P + P (*n* = 18), S-C (*n* = 19), S-P (*n* = 6)

In both generations, the F_1_ reproduction treatment influenced snail size. In the F_1_ generation, we found that size was influenced solely by reproduction treatment, with selfing snails being larger than outcrossing snails (Table 2; Fig. 2; Outcross: 2160.3 ± 37.9, Self: 2316.9 ± 63.5). In the F_2_ generation, size was influenced by F_1_ reproduction treatment as well as F_1_ predator treatment, but the impact of the predator treatment depended on whether or not it was the maternal or paternal snail that had experienced predation risk (Table 2; Fig. 3). Snails whose parents reproduced via selfing were smaller than snails whose parents reproduced via outcrossing (Outcross: 1415.5 ± 52.1, Self: 911.3 ± 46.1). For F_2_ snails produced via outcrossing, parental exposure to predation risk, especially paternal exposure to predation risk, resulted in larger snails (O-C + C: 1166.3 ± 93.1, O-C + P: 1578.8 ± 128.4, O-P + C: 1341.3 ± 78.7, O-P + P: 1643.6 ± 90.0; Fig. 3). F_2_ snails that were produced via selfing were smaller when their parent snail was exposed to predation risk (S-P: 821.4 ± 126.7, S-C: 941.2 ± 45; Fig. 3).

**Table 2:** Model selection results for F_1_ and F_2_ snail size based on combinations of F_1_ predator treatment (Pred or Maternal Pred and Paternal Pred; predator or control with Maternal and Paternal referring to the predator treatment of the snail that deposited the egg mass and the predator treatment of the snail that was paired with the egg-depositing snail) and F_1_ reproduction treatment (Repro; selfing or outcrossing)

Model	ΔAICc	Df	Weights
F_1_ size			
Repro	0.0	3	0.53
Repro + Pred	2.1	4	0.19
Null	2.9	2	0.13
Repro * Pred	3.1	5	0.11
Pred	4.9	3	0.05
F_2_ size			
Repro * Paternal Pred	0.0	5	0.72
Repro * Maternal Pred * Paternal Pred	1.9	7	0.27
Repro	13.0	3	0.001
Repro * Maternal Pred	13.1	5	0.001
Maternal Pred * Paternal Pred	25.3	5	< 0.001
Paternal Pred	28.3	3	< 0.001
Maternal Pred	34.2	3	< 0.001
Null	38.9	2	< 0.001

* Interaction terms are indicated with asterisks

For each model, centroid served as the response variable

**Fig. 4:**
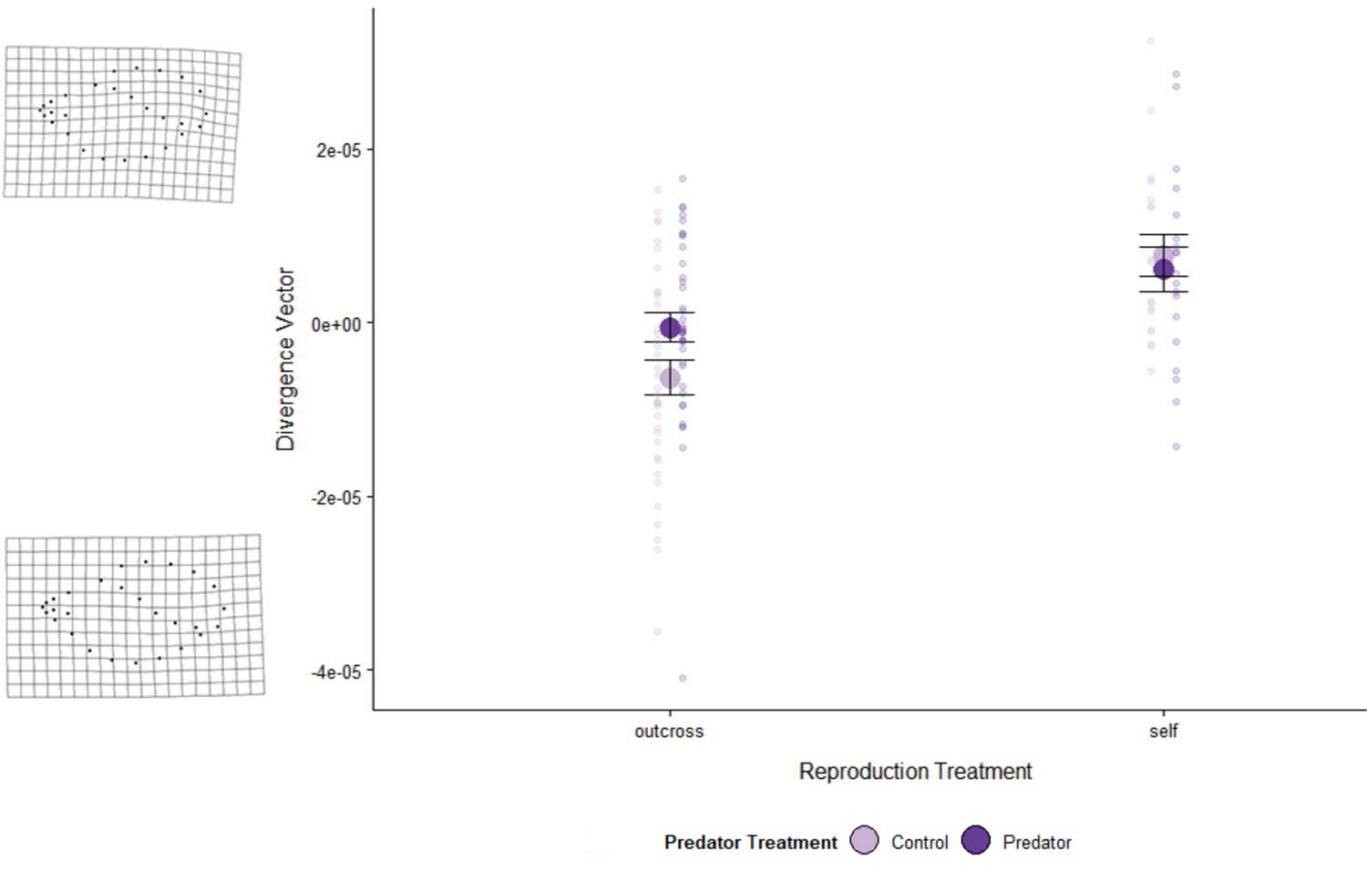
Mean divergence vector ± SE for two F_1_ reproduction treatments. F_1_ predator treatments: water-only control cues (light purple), predator cues (dark purple). The transparent purple points indicate individual snail data. The deformation grids represent the shape of snails that had higher or lower divergence vector values relative to mean snail shape (i.e., the upper deformation grid represents the shape of a selfing snail, while the lower deformation grid represents the shape of an outcrossing snail). Sample size by treatment combination: outcross-control (*n* = 37), outcross-predator (*n* = 39), selfing-control (*n* = 18), and selfing-predator (*n* = 19)

F_1_ treatments and size influenced F_1_ snail shape, but not F_2_ snail shape. On the whole, larger F_1_ snails had larger apertures (Centroid x RWs (19,812), F = 3.28, p < 0.001). After controlling for the effects of snail size, F_1_ reproduction treatment influenced F_1_ snail shape (Centroid x RWs x Repro Treatment (20, 861) = 2.35, p = 0.0008; Fig. 4) while F_1_ predator treatment did not (Centroid x RWs x Pred Treatment (20, 862) = 1.0, p = 0.45). The interaction between F_1_ predator and reproduction treatments, after controlling for the effects of size, was also significant (Centroid x RWs x Pred Treatment x Repro Treatment (20, 812) = 1.83, p = 0.01). In general, F_1_ snails that reproduced via selfing had narrower apertures than snails that reproduced via outcrossing and, of the snails that outcrossed, those that experienced predation risk had relatively narrower apertures (Fig. 4). For the F_2_ snails, shell shape was solely impacted by snail size (Centroid x RWs (19,614), *F* = 3.20, *p* < 0.001, all others *p* > 0.12).

### F_**2**_ anti-predator behavior

**Fig. 5:**
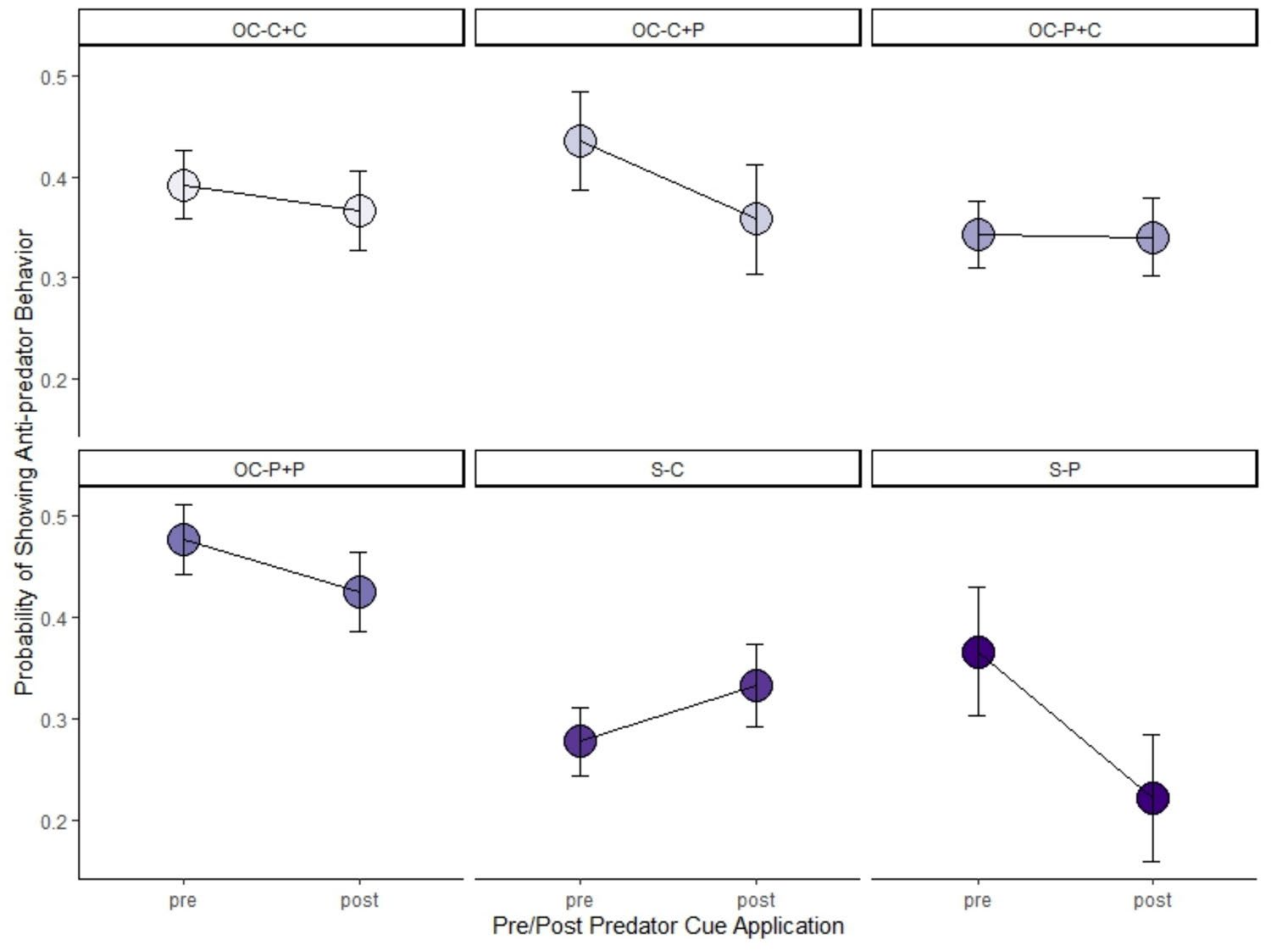
Mean probability of exhibiting crawling-out (i.e., anti-predator) behavior ± SE for snails in the six F_2_ treatments (before and after the addition of predator cues. F_2_ treatments are indicated as F_1_ reproduction treatment (S = selfing, OC = outcrossing)-F_1_ maternal predator treatment + F_1_ paternal predator treatment (C = control cues, P = predator cues). Sample size by treatment combination: OC-C + C (*n* = 17), OC-C + P (*n* = 9), OC-P + C (*n* = 17), OC-P + P (*n* = 18), S-C (*n* = 19), S-P (*n* = 6)

The most supported model to explain F_2_ snail behavior contained an interaction between before/after the addition of predator cues, F_1_ reproduction treatment, and the maternal and paternal F_1_ predator treatment (Table 3; Fig. 5). Overall, snails were less likely to exhibit anti-predator behavior after the addition of predator cues, especially snails whose parents were in the S-P treatment. The exception to this was the snails whose parents were in the S-C treatment; snails in the S-C treatment were more likely to exhibit anti-predator behavior after predator cues were added (Fig. 5).

**Table 3:** Model selection results for F_2_ snail behavior based on combinations of F_1_ predator treatment (Maternal Pred and Paternal Pred; predator or control with Maternal and Paternal referring to the predator treatment of the snail that deposited the egg mass and the predator treatment of the snail that was paired with the egg-depositing snail) and F_1_ reproduction treatment (Repro; selfing or outcrossing), and before/after the addition of predator cue (Cue)

Model	ΔAICc	df	Weights
Cue*Repro*Maternal Pred*Paternal Pred	0.0	13	0.86
Cue*Maternal Pred + Cue*Paternal Pred	3.99	7	0.12
Cue	8.21	3	0.01
Cue*Repro	9.77	5	0.01
Null	11.64	2	< 0.01
Repro	13.28	3	< 0.01
Maternal Pred	13.33	3	< 0.01
Paternal Pred	13.44	3	< 0.01
Repro*Maternal Pred*Paternal Pred	21.07	7	< 0.01

* Interaction terms are indicated with asterisks

For each model, individual snail was treated as a random effect

## Discussion

We found evidence that predation risk and reproductive strategy can impact the within- and transgenerational plasticity of Physid snails. Specifically, we found that F_1_ phenotype and reproductive activity were heavily influenced by our reproduction treatments with some additional impacts of predation risk on reproductive activity. Aspects of F_2_ snail phenotype, in contrast, were either jointly impacted by parental predation and reproduction treatments or did not express any transgenerational effects. On the whole, we found support that reproductive strategy can impact the transgenerational effects of predation risk.

Predator and reproduction treatments separately impacted the number of egg masses produced by the F_1_ generation. Predator-treated snails produced fewer egg masses than control-treated snails, and selfers produced fewer egg masses than outcrossers (Fig. 1). However, there was no significant difference in the number of eggs per mass. This would suggest that control-treated snails and snails that reproduced via outcrossing independently had higher reproductive success than predator-treated snails and snails that reproduced via selfing, respectively. Previous studies, without incorporating predation risk, found that snails that preferentially outcross, like the species used in this study, experience reduced fecundity when isolated from a mate (Jarne et al. 1991, 2000). Reduced fecundity is also a common non-consumptive effect associated with predation risk (Magnhagen 1991). However, it should be noted that previous studies spanning the entire lifetime of Physid snails did not find evidence of decreased fecundity associated with predation risk (Auld and Relyea 2008). We did not find evidence that our treatments significantly impacted egg viability. This is inconsistent with previous studies (e.g., Wethington and Dillon 1997), which found that selfing snails produced fewer fertile eggs and less viable offspring than snails outcrossed.

F_1_ snail shell size and shape were influenced by reproduction treatment. F_1_ snails that reproduced via selfing had larger shells than snails that reproduced via outcrossing (Fig. 3). In our reproductive activity data, we found that selfers produced fewer egg masses than outcrossers, and, therefore, may have been investing more energy toward growth rather than reproduction (Chase 1999; Tsitrone et al. 2003). After controlling for shell size, F_1_ shell shape was also impacted by reproduction treatment (Fig. 5). Snails exposed to predation risk may develop elongated shells (DeWitt et al. 2000) or narrower apertures to prevent predation by extraction (Alexander and Covich 1991). Alternatively, a narrow aperture observed may reflect a reduced growth rate (Hoverman and Relyea 2007, Handelsman et al. 2013). Both of these explanations, however, are inconsistent with our findings; snails that reproduced via selfing grew larger and had a narrower aperture than outcrossers, regardless of predation treatment. Given that the differences in shape were primarily at the aperture, it is possible that the act of intromission by the outcrossing snails or their relatively high reproductive output influenced their shell morphology, but future studies would have to be conducted to test this more thoroughly.

The F_2_ generation shell size was influenced by the reproduction and predator treatments of the F_1_ generation, while the F_2_ shell shape was not impacted by either treatment. Offspring of selfers were smaller than offspring of outcrossers, especially if their parent had experienced predation risk (Fig. 4). Similar to our results, Beaty et al. (2016) found that offspring of predator-exposed parents had larger shells; however, we did not find support for this pattern if the snails were produced by selfing. Many species experience decreased growth with increased homozygosity (Mitton 2000), which has previously been observed in the marine snail, *Crepidula convexa* (Li and Pechenik 2007). Additionally, our results from the F_1_ generation suggest that selfers were likely investing more in their own growth rather than in their offspring, likely contributing to the smaller average size of F_2_ snails from selfing parents. Within the outcrossed offspring, snails with predator-treated fathers were larger than those with control-treated fathers. This is contrary to expectations that size would be mediated by maternal effects because offspring investment is regulated by the mother to the eggs. Goeppner et al. (2023) found that snails with predator-exposed mothers were heavier though this response was lost when the fathers were also exposed to predation risk. To date, ours is the first study to report paternal effects on size in Physids. However, paternal effects on Physid behavior have been previously documented (e.g., Tariel et al. 2020a).

F_1_ reproduction and predator treatments interacted to influence the anti-predator behavior of F_2_ snails. We found that all F_2_ snails, except for S-C, were less likely to exhibit crawling-out behavior after adding predator cues (Fig. 5). This is in contrast to several previous studies, which found evidence of predator-induced transgenerational plasticity in the crawling-out behavior of Physids (Luquet and Tariel 2016; Tariel et al. 2020a, 2020b). However, Beaty et al. (2016) found no evidence of predator-induced transgenerational plasticity of this escape behavior. We scored our test based on crawling-out behavior, however, and it is possible the snails in our study were utilizing a different avoidance behavior (e.g., seeking refuge or burying in the substrate), as seen in other studies (e.g., Snyder 1967; Turner 1996). Thus, all F_2_ snail treatments may have been more likely to exhibit these alternative anti-predator behaviors. However, the S-C-treated F_2_ snails were more likely to exhibit crawling-out behavior after exposure to predator cues. This may be due to the fact that this group received information about the environment from one parental source. In other words, there was greater information certainty about the lack of predators in the environment, thus causing the snails to react so strongly to cues indicating predation risk. We saw no sex-specific effects on escape behavior in our study. However, Tariel et al. (2020a) found that when only the mother was exposed to predation risk, there was a negative effect on escape behavior. Furthermore, when both mother and father were exposed, paternal cues mitigated the negative effect of the maternal cues if the offspring were raised in control conditions. This demonstrates a complex pattern of cue integration in *Physa*, which is likely further complicated by selfing and outcrossing.

With the exception of the O-P + P treatment, F_2_ snails from predator-treated fathers (i.e., S-P and O-C + P) experienced high mortality. In previous work with Physid snails, snails that were produced via selfing, including groups exposed to predators and groups that were not exposed to predators, experienced decreased survival in comparison to snails produced by outcrossing (Auld 2010). However, this result may also be related to the paternal effect on size observed in our study. The O-C + P group experienced high mortality, but the survivors were large. This may be due to a trade-off between growth and survival. Prey may choose to inhabit areas with poor food sources to avoid predation (Lima 1998) as a result of paternally-transmitted information, which may, in turn, make them more vulnerable to death.

Overall, we found evidence that reproductive strategy (i.e., selfing or outcrossing) influences *Physa* transgenerational responses to predation risk. Our results show that morphology and behavior can be influenced across generations by predation risk and reproduction strategy, revealing the significance of epigenetic information certainty as it applies to anti-predator strategies. Offspring must consolidate information from parental source(s) and environmental sources to elicit plastic responses to predation risk. These results suggest a complex interaction between reproductive strategy, parental information cues, and current environmental cues when deciding how to respond to risk.

## Supplementary Information

Below is the link to the electronic supplementary material.

Supplementary file1 (DOCX 187 KB)

## Data Availability

After formal acceptance, the data will be deposited in Dryad, and the appropriate reference number will be provided.
