# Supplementary material for: Effects of selfing and outcrossing on transgenerational responses to predation risk
Source: Oecologia. 2025 May 30;207(6):94. doi: 10.1007/s00442-025-05729-w (PMC12125135; doi:10.1007/s00442-025-05729-w)
Supplement: Supplementary file 1 — Supplementary file1 (DOCX 187 KB) [file 442_2025_5729_MOESM1_ESM.docx]

**Title –** Effects of Selfing and Outcrossing on Transgenerational Responses to Predation Risk

Haley R. Altadonna and Lynne E. Beaty

School of Science, Penn State Erie, The Behrend College, Erie Pennsylvania 16563

**Corresponding author**:

Lynne Beaty

*Email*: lzb345@psu.edu

*Phone:* (814) 898-7132

**Author contributions:** LEB conceived and designed the experiment, carried out data collection and data analysis, and revised the manuscript. HRA carried out data collection, and drafted and revised the manuscript. Both authors approved the manuscript for submission.


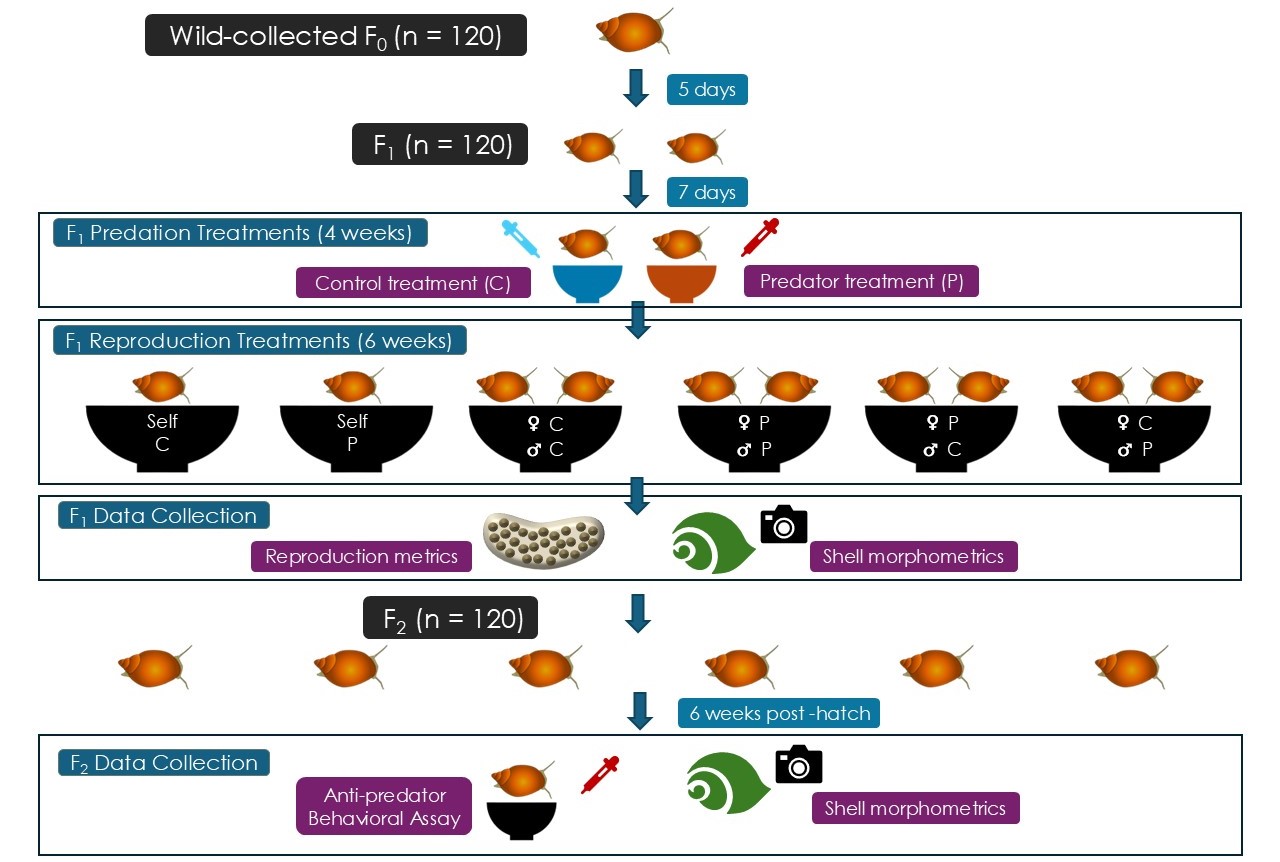


**Figure S1**: Graphical representation of the experimental design and timeline.
